# Worksite Influences on Obesogenic Behaviors in Low-Wage Workers in St Louis, Missouri, 2013–2014

**DOI:** 10.5888/pcd12.140406

**Published:** 2015-05-07

**Authors:** Jaime R. Strickland, Galen Pizzorno, Anna M. Kinghorn, Bradley A. Evanoff

**Affiliations:** Author Affiliations: Galen Pizzorno, Anna M. Kinghorn, Bradley Evanoff, Washington University School of Medicine, St. Louis, Missouri.

## Abstract

**Introduction:**

More than one-third of US adults are obese. Workplace programs to reduce obesity and improve overall health are not available or accessible to all workers, particularly low-wage workers among whom obesity is more prevalent. The goal of the study was to identify modifiable workplace factors and behaviors associated with diet and exercise to inform future workplace interventions to improve health.

**Methods:**

We distributed paper and online surveys to 2 groups of low-wage workers, hospital workers and retail sales workers, at the worksites. The surveys assessed obesity, obesogenic behaviors, workplace factors, and worker participation in workplace health programs (WHPs). Descriptive and regression analyses were conducted to examine workplace factors associated with obesogenic behaviors.

**Results:**

A total of 529 surveys were completed (219 hospital workers and 310 retail workers). More than 40% of workers were obese and 27% were overweight. In general, workers had poor diets (frequent consumption of sugary and high-fat foods) and engaged in little physical activity (only 30.9% met recommended physical activity guidelines). Access to and participation in workplace health programs varied greatly between hospital and retail sales workers. We identified several modifiable workplace factors, such as food source and work schedule, that were associated with diet, exercise, or participation in workplace health programs.

**Conclusion:**

This study illustrates the high prevalence of obesity and obesogenic behaviors workers in 2 low-wage groups. The differences between work groups indicated that each group had unique facilitators and barriers to healthy eating and exercise. An understanding of how socioeconomic, demographic, and work-related factors influence health will help to identify high-risk populations for intervention and to design interventions tailored and relevant to the target audiences.

## Introduction

More than one-third of US adults are obese (1), and obesity is a major contributor to increased medical costs and lost productivity (2–4). Obesity is associated with low income and education, even after controlling for other risk factors (4,5). Even modest weight loss is associated with improved health outcomes for such conditions as diabetes (6,7), and many evidence-based guidelines now recommend lifestyle interventions for weight management and disease prevention (8,9). Worksite wellness programs that incorporate weight management interventions are becoming more common (3) and can be an effective means of reaching low-wage populations (4,10,11).

Low-wage workers have less access to workplace wellness programs and are less likely to use them, creating an overlooked health disparity (10,12,13). Furthermore, low-wage jobs often entail shift work, irregular schedules, and little autonomy over work schedule (2), which may contribute to obesogenic behaviors, yet most existing worksite programs do not address such workplace factors (3,14–16). Understanding how the workplace influences obesity and how existing structures can be used to change behavior will inform the development of more effective wellness programs to target obesity and reduce health disparities (3,10).

This study examined some workplace determinants of obesogenic behaviors in 2 groups of low-wage workers. Additionally, we examined factors related to participation in existing workplace health programs (WHPs). The goal of the study was to identify modifiable workplace factors and behaviors associated with diet and exercise to inform future workplace interventions to improve health.

## Methods

### Study population and recruitment

We worked with a large health care system and 2 local chapters of a national union representing retail workers to recruit participants. The health care system and the union represent large, fast-growing segments of the low-wage workforce, and both expressed interest in improving their workplace wellness efforts. Workers were recruited and surveyed from November 2013 through June 2014. We targeted hospital departments with high proportions of low-wage workers, including housekeepers, food service workers, patient care technicians, and unit secretaries; retail workers were primarily employed by 3 regional retail chains. We attempted to recruit all workers within targeted departments, stores, or union meetings and worked with supervisors, store managers, and union leaders to distribute paper surveys packets. Packets included a recruitment letter, consent form, and survey. Participants could return paper surveys in person to a research team member at a specified time and location or by mail using a prepaid envelope; they were compensated for their time. A small number of surveys were offered online to hospital employees who did computer work. All participants were at least 18 years of age and spoke English. This study was approved by the Washington University Institutional Review Board.

### Survey development and administration

The survey assessed various domains including demographics, job characteristics, and work environment (eg, schedule, wages, social support, employer’s value of workers’ health), availability of and participation in WHPs, health behaviors (eg, diet, physical activity, willingness to change health behaviors), and health status (eg, height, weight, overall health, health conditions). To measure the 3 primary outcomes of diet, physical activity, and participation in WHPs, we used well-established survey tools: the Rapid Eating Assessment for Participants Short Version (REAP-S) (17), the 2-question physical activity assessment (18), and 16 items from the Worksite and Energy Balance Survey (WEBS) to measure availability and participation in WHPs (such as health fairs, exercise programs and facilities, flexible time for physical activity, and incentives to walk or bike to work) (19). The survey also included the SF-8 to measure health status (20), the Supervisor and Coworker Support scales from the Job Content Questionnaire (21), questions from the WEBS survey to determine food source at work, a question from the National Health and Nutrition Examination Survey Occupation Questionnaire Section (22) to assess work schedule, and a revised version of the Stanford Brief Activity Survey to determine physical activity at work (23).Willingness to change eating behaviors was measured by using a question from the REAP-S; we created a similar question for physical activity. Prior to distribution, we conducted pilot testing of the survey among both hospital and retail workers to ensure clarity, relevance, and readability. The survey took approximately 15 to 20 minutes to complete.

### Data analysis

Body mass index (BMI) (weight in kg/height in m^2^) was calculated by using self-reported height and weight. Aggregate scores for the Job Content Questionnaire and the SF-8 physical and mental well-being scores were calculated by using published procedures (20,21). To assess food sources at work, participants reported the number of days that they brought food from home, purchased food at their workplace, or purchased takeout food to eat at work. Since workers often brought and purchased food on the same day, we assessed food sources in 2 ways: 1) we categorized the primary food source as the source of food more than 60% of the time, and 2) we calculated the proportion of time workers used each source. Work groups were compared using Pearson χ^2^ and analysis of variance (ANOVA); significance was assessed at *P* ≤.05.

We examined possible predictors of 3 outcomes: diet, exercise, and WHP participation. To assess diet, we used the REAP-S total score (17), which reflects how often a participant engages in healthy and unhealthy eating behaviors. Scores ranged from 13 to 39 with a lower score indicating healthier behaviors. We also examined REAP-S subscores for consumption of fatty foods and sugary foods. To categorize people as either meeting or not meeting the recommended level of exercise (24), we estimated total physical activity minutes per week on the basis of answers to the 2 physical activity questions. Participation and availability of WHPs was calculated as a positive response for participation in any 1 of the 16 programs queried.

Student’s *t* test was conducted on dichotomous predictors of REAPS-S total score, and Spearman correlations were conducted for interval and ordinal predictors. Univariate logistic regression was conducted for predictors of exercise and WHP participation, yielding odds ratios and 95% confidence intervals. For each outcome, significant predictors (*P* ≤.05) in the univariate analyses were included in multivariate models. We analyzed the REAP-S total score by using multivariate ordinary least squares regression and used multivariate logistic regression to analyze exercise and WHP participation. Analyses were conducted using IBM SPSS version 20 and R version 3.1.0 (IBM Corporation).

## Results

A total of 219 hospital workers (30.0% response rate) and 310 retail workers (57.5% response rate) completed the survey. The median wage was $11.26 per hour; 46% of respondents had an annual household income below $30,000 (Table 1). Mean BMI was 29.5 (standard deviation [SD], 7.2), 67.8% had a BMI at or above 25, and 41.1% were obese (BMI ≥30), which was above the national prevalence of 34.9% for 2011–2012 (1). Nearly half of respondents reported having 1 or more of the following diseases: hypertension, arthritis, high cholesterol, or diabetes. Mental and physical health, as shown by the SF-8 scores, were slightly worse than values for the general US population (25).

**Table 1:** Demographics, Health Status, and Potential Predictors of Obesogenic Behaviors in Low-wage Hospital and Retail Workers, St. Louis, Missouri, 2013–2014

Demographics	All Workers (n = 529)^a^	Hospital Workers (n = 219)^a^	Retail Workers (n = 310)^a^	*P* Value
Age, mean (SD), y	43.0 (14.9)	41.8 (13.9)	43.8 (15.5)	.14
Female	66.0	76.7	58.4	<.001
Racial/ethnic minority	50.6	63.6	41.4	<.001
Some college	58.8	58.7	58.9	.97
Hourly wage, median, $	11.26	11.00	11.70	.87
Household income < $30,000/y	46.5	56.9	39.1	<.001
**Health status**				
BMI, mean (SD), kg/m^2^	29.5 (7.2)	30.5 (7.6)	28.7 (6.9)	.005
Normal weight (BMI<25.0)	32.2	27.4	35.5	.05
Overweight (BMI 25.0–29.9)	26.7	21.9	30.2	.03
Obese (BMI ≥30.0)	41.1	50.7	34.2	<.001
Current smoker	16.6	12.8	19.3	.05
SF-8 physical score, mean (SD)	49.1 (8.4)	48.7 (8.6)	49.3 (8.3)	.39
SF-8 mental score, mean (SD)	49.0 (10.3)	49.5 (10.3)	48.7 (10.3)	.38
Diabetes	9.8	12.3	8.1	.11
Hypertension	21.9	25.6	19.4	.09
High cholesterol	17.0	18.7	15.8	.38
Arthritis	21.0	23.3	19.4	.27
Have ≥1 conditions listed above^b^ or other diseases	48.0	49.3	47.1	.62
Have ≥2 conditions listed above^b^ or other diseases	21.7	24.7	19.7	.17
Missed work because of health problem in last 4 weeks	13.2	15.5	11.5	.18
**Diet**				
REAP-S score, mean (SD)	25.5 (4.5)	25.0 (4.5)	25.9 (4.5)	.03
Often consume sugary drinks and/or sweets	45.8	39.7	50.0	.02
Often eat fatty foods	55.5	55.6	55.4	.96
Bring food from home	23.3	27.1	20.6	.08
Buy food at work	38.8	36.9	40.2	.45
Do not eat regularly at work	13.1	8.4	16.3	.008
**Activity level**				
Get recommended level of exercise	30.9	40.8	35.5	.22
Sit or stand at work	35.7	29.0	40.3	.009
**Work environment**				
Hours worked per week, mean (SD)	36.8 (9.5)	38.9 (7.6)	35.3 (10.3)	<.001
Nonday shifts	55.8	45.4	63.2	<.001
Irregular shifts	32.4	6.0	51.3	<.001
Supervisor and Coworker Support scales score (21), mean (SD)	23.3 (4.3)	22.9 (4.7)	23.6 (3.9)	.06
Company values worker health	78.7	85.4	74.1	.002
One or more WHPs offered	66.9	92.2	49.0	<.001
Participated in ≥1 WHPs	54.8	73.8	29.6	<.001

Abbreviations: BMI, body mass index; REAP, Rapid Eating Assessment for Participants Short Version; SD, standard deviation; WHP, workplace health program.

a Values are percentages unless otherwise noted.

b Conditions included are diabetes, hypertension, high total cholesterol, and arthritis.

### Obesogenic behaviors

The overall population had a REAP-S total score of 25.5 (SD, 4.5), indicating that many respondents had unhealthy eating habits. Compared with nonobese participants, obese participants had significantly higher scores on the REAP-S (25.1 vs 26.0, *P* = .04) and the fatty foods subscale (8.0 vs 8.4, *P* = .047); there was no significant difference in the sugar subscale. The source of food at work varied greatly; about 23.3% primarily brought from home, 38.8% primarily bought food at work, 24% split between bringing and buying, and 13.1% did not regularly eat at work.

Overall, only 30.9% reported getting the recommended level of exercise, lower than the 46.1% found in a national sample (26). Obese workers were less likely to get recommended levels of exercise than nonobese workers (23.8% vs 35.8%, *P* = .006). More than one-third (35.7%) reported spending most of their work day either sitting or standing, whereas 28.7% said they spend most of the day walking, 28.9% said they spend most of the day lifting or pushing heavy objects or moving most of their body, and 4.3% said they do hard physical labor most of the day.

Most participants reported willingness to change both eating habits and physical activity to be healthier (reporting at least a 4 on a scale of 1 to 5 with 1 being “not at all willing” and 5 being “very willing”); many said they had already changed eating patterns or physical activity in the last year because of health concerns.

### Work environment

More than half (55.8%) of respondents did not regularly work day shifts, and 32.4% reported working irregular schedules. Overall, participants felt that their supervisors and coworkers were supportive as indicated by high Supervisor and Coworker Support scales scores. Additionally, most workers agreed or strongly agreed that their companies valued healthy workers. Participation in any WHP was 36.7%; among those who reported that WHPs were offered, the participation rate was 54.8%. Availability of WHPs was not associated with lower rates of obesity, but those who participated in 1 or more programs were less likely to be obese than those who did not (49.7% vs 60.7%).

### Predictors of healthy diet

In univariate analyses, a lower REAP-S score, (ie, healthier diet) was associated with older age, higher wages, greater number of hours worked, higher rate of bringing food from home, having some college education, participating in a WHP, and working for the hospital system rather than for retail stores (Table 2). Minority status, nonday shifts, irregular shifts, and higher rates of buying food at work or getting takeout were associated with unhealthier diet. The final multivariate models had a *R^2^* value for the REAP-S total score of 0.24 for all workers (Table 3). Bringing food from home was the strongest predictor of healthy diet for all workers. Older age, lower wages, nonminority status, some college education, and participation in a WHP were also predictors of healthy diet.

**Table 2:** Univariate Results for Predictors of Diet, Exercise, and Participation in Workplace Health Programs Among Low-wage Hospital and Retail Workers,^a^ St. Louis, Missouri, 2013–2014

Predictor	Diet (REAP-S Score) (n = 529)	Recommended Exercise Level (n = 529)	Participated in 1 or More WHPs (If Offered) (n = 354)
	Spearman *r* (*P* Value)	Logistic Regression Odds Ratio (*P* Value)	Logistic Regression Odds Ratio (*P* Value)
Age	−0.19 (<.001)^b^	0.98 (.004)^b^	0.98 (.01)^b^
Wage	−0.17 (.001)^b^^,^ c	0.97 (.13)	1.00 (.96)^b^
Hours worked per week	−0.14 (.002)^b^	1.00 (.74)^c^	1.02 (.16)
Social support at work	0.03 (.57)	1.04 (.14)	1.01 (.71)
Bring food from home, rate	−0.33 (<.001))^b^^,^ c	2.01 (.02^c^	1.16 (.65)
Buy food, rate	0.28 (<.001)^b^^,^ c	0.49 (.02)^c^	0.94 (.84)
Buy takeout, rate	0.13 (.006)^c^	.98 (.98)	0.62 (.54)
**Difference in mean score (*P* Value)**			
Female	−0.73 (.09)^c^^,^ d	0.67 (.051)	1.68 (.02)
Racial/ethnic minority	2.13 (<.001)^b^^,^ c ^,^ d	1.42 (.08)	2.11 (.001)^b^
Some college	−1.09 (.01)^c^^,^ d	1.18 (.40)	0.82 (.37)
Hospital worker	−0.9 (.03)^d^	1.26 (.24)	6.69 (<.001)
Nonday shifts	0.98 (.02)^d^	1.25 (.27)	0.48 (.001)
Irregular shifts	0.94 (.03)^d^	0.77 (.22)	0.24 (<.001)
Physical activity at work	0.57 (.12)^d^	1.82 (.01)^b^	1.17 (.49)
Company values health	−0.65 (.19)^d^	1.42 (.16)	1.27 (.39)
WHP offered	−0.82 (.06)^d^	1.10 (.64)	NA
Participated in WHP	−1.32 (.002)^b^^,^ d	1.79 (.004)^b^	NA

Abbreviations: NA, not applicable; REAP-S, Rapid Eating Assessment for Participants Short Version; WHP, workplace health program.

a Numbers represent both worker groups.

b Significant predictors among retail workers (*P* ≤.05).

c Significant predictors among hospital workers (*P* ≤.05).

d Difference in mean REAP-S scores between dichotomous categories

**Table 3:** Multivariate Regression Results for Predictors of Diet, Exercise, and Participation in Workplace Health Programs, St. Louis, Missouri, 2013–2014

Predictors	Value
**Diet^a^**	
Age	−0.05 (.006)
Wage	0.09 (.03)
Hours worked per week	−0.03 (.29)
Bring food from home, rate	−3.43 (<.001)
Buy takeout food, rate	−1.96 (.32)
Racial/ethnic minority	2.27 (<.001)
Some college	−0.91 (.04)
Hospital worker	−0.10 (.85)
Nonday shift	0.51 (.27)
Participated in WHP	−1.44 (.006)
**Exercise^b^**	
Age	0.98 (0.97–1.00)
Buy food, rate	0.66 (0.13–3.40)
Bring food from home, rate	1.73 (0.34–8.85)
Physical activity at work	1.69 (1.06–2.72)
Participated in WHP	1.67 (1.08–2.60)
**Participation in WHP^b^**	
Age	0.98 (.096–0.99)
Female	1.09 (0.63–1.89)
Racial/ethnic minority	1.73 (1.06–2.85)
Hospital worker	5.09 (2.77–9.38)
Nonday shifts	0.67 (0.37–1.22)
Irregular shifts	0.79 (0.36–1.71)

Abbreviation: WHP, workplace health program.

a Predictors of diet according to Rapid Eating Assessment for Participants Short Version total score; values are unstandardized coefficients (*P* value); *R* ^2^ = 0.24.

b Values are odds ratio (95% confidence interval).

### Predictors of exercise

Fewer predictors of exercise were found via univariate analysis (Table 2). For all workers, younger age, higher rate of bringing food, lower rate of buying food, having more physical activity at work, and participating in WHPs were all significant predictors of exercise. In the multivariate logistic regression model, only physical activity at work and WHP participation were significant predictors of exercise for all workers (Table 3).

### Predictors of WHP

Univariate logistic regression analyses of the 354 workers who indicated that their company offered 1 or more WHPs showed that younger age, being female, being a minority, and working for the hospital predicted WHP participation, whereas working nonday shifts and having irregular schedules were associated with nonparticipation (Table 2). In the multivariate model for all workers, younger age, minority status, and being a hospital worker predicted participation in WHP.

### Group differences

Several differences between the work groups may inform future interventions. Table 1 shows comparisons between hospital workers and retail workers. Retail workers were more likely to work nonday shifts, have irregular schedules, and sit or stand in 1 place most of the day compared with hospital workers (Table 1). Hospital workers were more likely to believe that their company valued healthy workers than retail workers and also reported greater availability of WHPs. Significant (*P* ≤.05) univariate associations with diet, exercise, and WHP participation for hospital workers and retail workers are noted in Table 2.

The *R^2^* values for the multivariate models predicting the overall REAP-S score were 0.26 for hospital workers, and 0.22 for retail workers. For hospital workers, bringing food from home and nonminority status were associated with a healthier diet; bringing food from home, participation in a WHP, younger age, and nonminority status were predictors of healthier diet in retail workers (Table 3).

In the multivariate models, WHP participation was the only significant predictor of exercise in retail workers (odds ratio [OR] 2.16, *P* = .03); there were no significant predictors of exercise for hospital workers. Although participation in any WHP was not a significant predictor of exercise in hospital workers, participation in 4 programs was associated with exercise in these workers: workplace exercise programs (OR, 3.12; *P* = .04), reduced price gym memberships (OR, 4.30; *P* = .008), signs encouraging the use of stairs (OR, 4.35; *P* = .02), and brochures or a poster encouraging healthy behaviors (OR, 3.00; *P* = .009).

## Discussion

Our study group of low-wage workers had slightly poorer health than the general US population, but this is probably typical of low-wage American workers. Obesogenic behaviors such as a diet high in fat and infrequent exercise were common and were associated with poor health outcomes (ie, high rates of obesity and illness). Despite their obesogenic behaviors, most workers indicated they were willing to change their diet and exercise habits to be healthier. Employer or union-based interventions may help workers achieve their desired behaviors and healthy weight.

We identified several modifiable workplace factors associated with diet. Food source was the strongest predictor of diet; bringing food from home more often was associated with healthier eating, whereas buying food at the worksite was associated with unhealthy eating. Preparing food ahead of time may allow workers to plan healthy food options rather than making spontaneous, unhealthy purchases when they are hungry or have little time. Additionally, bringing food from home may help with portion control, as cafeteria or restaurant food is often sold in large portions. Employers can encourage workers to bring their own food to work by providing microwave ovens and refrigerators, organizing healthy potlucks, and offering suggestions for healthy recipes and tips for easy meal planning. Alternatively, employers could provide healthier food options for purchase that are highly visible, readily available, and low in cost. Irregular work schedules, nonday shifts, and nonparticipation in a WHP were also predictors of unhealthy diet; these factors are all potential targets for interventions.

Consistent with previous findings, our results indicated that participating in a WHP was associated with more exercise outside of work (11); greater physical activity at work was also a predictor of meeting weekly exercise recommendations. This study had limited power to detect significant predictors for each work group. However, 1 difference is worth noting: hours worked had opposite associations for the 2 work groups. Working more hours per week was positively associated with exercise in hospital workers, but negatively associated in the retail group, though this association was not significant for retail workers. Schedule regularity may partly explain this finding, because many of the retail workers in this study reported having irregular schedules. Among workers with irregular schedules, those who met the recommended guidelines for exercise worked fewer hours than those who did not (32.6 h vs 36.1 h, *P* = .03); there were no significant association in workers with regular schedules. Thus, it may be the combination of irregular schedules and longer work hours that interferes with exercising. Additionally, irregular schedules may influence the ability to plan ahead and maintain diet and exercise routines. Irregular and unpredictable work schedules are becoming more common for retail workers, imposing a particular burden on low-wage workers (27). Future research should examine the health implications of irregular schedules.

Differences observed between hospital workers and retail workers highlight the complexity of obesity and behavior change as well as the need for tailored approaches to workplace health programs. Designing programs that are tailored to the needs of employees may result in greater reach and adoption of interventions, ultimately producing behavior change. One way of creating interventions that are relevant to a work group is a participatory approach, in which workers provide input into the types of interventions that would be useful and appealing to them. This approach has been successful in safety and ergonomic interventions but little studied or tested for workplace health behavior interventions (28). Socioeconomic and demographic factors play a strong role in health behaviors and health status. Although employers can target WHP efforts to high-risk populations, improvements in health among low-wage workers may ultimately require more systematic changes, such as better pay and benefits, more regular work schedules, and compensated time for participation in health activities (29).

Our study has several limitations. First, response rates in both groups were low because of limitations in our recruitment and follow-up methods. Some managers allowed us to talk to workers directly, but most would only distribute the survey and reminders on our behalf. Second, all data were self-reported by the workers and may be subject to poor recall or social desirability bias. Questions regarding WHP offerings measured workers’ awareness of the availability of these programs. Retail workers’ reports of few WHP offerings were generally accurate; hospital workers had more available WHPs but were often unaware of programs that were available to them. Improved communication may be effective in increasing program awareness and, eventually, participation. Third, the REAP-S scale was designed for use in clinical settings rather than in general population studies. We chose this measure because it is brief, designed for lower literacy people (17), and assesses compliance with dietary guidelines. Although it is more limited than longer dietary questionnaires, it measures specific healthy and unhealthy behaviors that could be targeted for intervention. Similarly, we chose 2 simplified questions measuring exercise so as not to burden participants with a lengthy survey. Consequently, we found few significant predictors of exercise, which may be a result of using an insensitive measure. Fourth, our brief survey did not ask about many important risk factors for obesity and obesogenic behaviors; some of these factors were explored in a qualitative analysis of these populations that is reported separately (30). Finally, some of our study findings are probably industry-specific and may not be generalizable to other low-wage populations.

In summary, our study highlights the high prevalence of obesity and obesogenic behaviors among 2 low-wage worker groups and describes workplace influences on healthy behaviors. Between-group differences suggest that interventions should be tailored to different worker groups. From these results, we recently started an intervention based on the Healthy Workforce Participatory Program (31) in a retail store we worked with in this project. We will use previous qualitative data (Strickland et al, unpublished data, August 2014) and results from this study to inform a participatory worker group intervention that will elicit worker input for changes at the worksite to support healthy behaviors.
